# *Ficus deltoidea* ameliorates biochemical, hormonal, and histomorphometric changes in letrozole-induced polycystic ovarian syndrome rats

**DOI:** 10.1186/s12906-021-03452-6

**Published:** 2021-11-29

**Authors:** Muhammad Aliff Haslan, Nurdiana Samsulrizal, Nooraain Hashim, Noor Syaffinaz Noor Mohamad Zin, Farshad H. Shirazi, Yong Meng Goh

**Affiliations:** 1 Faculty of Applied Sciences, Universiti Teknologi MARA, 40450 Shah Alam, Selangor Malaysia; 2 Department of Pharmacology and Toxicology, School of Pharmacy, Shahid Beheshti University of Medical Sciences, Tehran, Iran; 3 Pharmaceutical Sciences Research Center, Shahid Beheshti University of Medical Sciences, Tehran, Iran; 4 Department of Veterinary Preclinical Sciences, Faculty of Veterinary Medicine, Universiti Putra Malaysia (UPM), Serdang, Malaysia

**Keywords:** Polycystic ovarian syndrome, *Ficus deltoidea*, Letrozole, Lipid, Antioxidant, Ovary, Uterus

## Abstract

**Background:**

Insulin resistance and hormonal imbalances are key features in the pathophysiology of polycystic ovarian syndrome (PCOS). We have previously shown that *Ficus deltoidea* var. *deltoidea* Jack (Moraceae) can improve insulin sensitivity and hormonal profile in PCOS female rats. However, biological characteristics underpinning the therapeutic effects of *F. deltoidea* for treating PCOS remain to be clarified. This study aims to investigate the biochemical, hormonal, and histomorphometric changes in letrozole (LTZ)-induced PCOS female rats following treatment with *F. deltoidea*.

**Methods:**

PCOS was induced in rats except for normal control by administering LTZ at 1 mg/kg/day for 21 days. Methanolic extract of *F. deltoidea* leaf was then orally administered to the PCOS rats at the dose of 250, 500, or 1000 mg/kg/day, respectively for 15 consecutive days. Lipid profile was measured enzymatically in serum. The circulating concentrations of reproductive hormone and antioxidant enzymes were determined by ELISA assays. Ovarian and uterus histomorphometric changes were further observed by hematoxylin and eosin (H&E) staining.

**Results:**

The results showed that treatment with *F. deltoidea* at the dose of 500 and 1000 mg/kg/day reduced insulin resistance, obesity indices, total cholesterol, triglycerides, low-density lipoprotein cholesterol (LDL), malondialdehyde (MDA), testosterone, luteinizing hormone (LH), and follicle-stimulating hormone (FSH) to near-normal levels in PCOS rats. The levels of high-density lipoprotein cholesterol (HDL), estrogen, and superoxide dismutase (SOD) are also similar to those observed in normal control rats. Histomorphometric measurements confirmed that *F. deltoidea* increased the corpus luteum number and the endometrial thickness.

**Conclusions:**

*F. deltoidea* can reverse PCOS symptoms in female rats by improving insulin sensitivity, antioxidant activities, hormonal imbalance, and histological changes. These findings suggest the potential use of *F. deltoidea* as an adjuvant agent in the treatment program of PCOS.

## Background

Polycystic ovarian syndrome (PCOS) is a chronic endocrine disorder that occurs in women of reproductive age. It is estimated to affect up to 4–12% of women worldwide [1]. PCOS often develops due to an imbalance of reproductive hormones and insulin resistance. The major clinical features of PCOS include ovarian cysts, irregular menstrual cycles, weight gain, and fertility problems [2]. The formation of ovarian cysts can interrupt ovulation and eventually leads to infertility. It is therefore not surprising that the prevalence of infertility in women with PCOS is about 70 to 80% [3]. PCOS has also been found to increase the risk of miscarriage, anxiety, and depression [4].

Metformin and clomiphene citrate are the common drugs prescribed for women with PCOS [5]. Treatment with metformin and clomiphene citrate improved ovulation and pregnancy rates of infertile patients with PCOS [6]. However, these treatments are significantly less effective in obese women with PCOS. It is important to note that about 40–60% of women with PCOS are overweight or obese [7]. Metformin and clomiphene citrate treatment are also associated with several adverse effects such as diarrhea, nausea, vaginal/uterine bleeding, breast tenderness, hot flashes, and abdominal pain [8].

Plants have been used for decades to address human fertility issues, and one of them is *Ficus deltoidea* or also known as Mas Cotek in Malaysia. *F. deltoidea* is growing wild in Kelantan, Terengganu, Sabah, Sarawak, and Kalimantan. It has recently been formulated, packaged, and distributed as a tonic tea, tea, and capsules across countries, in addition to being boiled in water for consumption [9]. The methanolic extract of *F. deltoidea* leaf has been reported to be rich in tannins, alkaloids, saponins, phenols, flavones, isoflavones, and flavonoids [10]. The presence of these compounds is beneficial for treating dyslipidemia, diabetes, heart disease, cancer, and infertility cases such as PCOS [11]. The antihypertensive [12], chemopreventive, and chemotherapeutic [13] activities of *F. deltoidea* have indeed been reported in animal studies*. F. deltoidea* also improved depressive behavior in rats [14]. Our previous studies have demonstrated that *F. deltoidea* is not only capable of promoting fertility in diabetic male rats [15] but also improving hormonal balance in PCOS female rats [16]. We have also shown that *F. deltoidea* increased cognitive performance [17] and attenuated tissue morphology changes by increasing antioxidant activities in diabetic rats [18, 19]. To the best of our knowledge, despite past extensive studies, the therapeutic activity of *F. deltoidea* on PCOS rats has not yet been fully elucidated. We presently examine the effects of *F. deltoidea* on the hormonal profile, biochemical parameters, and histological changes in LTZ-induced PCOS in rats to address these gaps in knowledge.

## Methods

### Sample collection and identification

*F. deltoidea* leaves were purchased from Moro Seri Utama Enterprise, Batu Pahat, Johor, Malaysia in September 2016. Following taxonomic authentication, the leaves sample was deposited at the Herbarium Unit, Universiti Kebangsaan Malaysia, Bangi with a voucher specimen (UKMB40315).

### Preparation of *F. deltoidea* methanolic extract

The leaves of *F. deltoidea* were cleaned with running tap water and air-dried in an air oven at 40 °C. The dried leaves were then ground to obtain a fine brownish powder [20]. For extraction, the powder was macerated with 1 L of absolute methanol (95%) for three days at 27 °C with a ratio of 1:10 [21]. The liquid extract was evaporated under reduced pressure at 40 °C using a rotary evaporator (Buchi, Switzerland). The dried extract was divided into smaller batches in tightly closed glass jar and stored at − 20 °C until further use to reduce moisture and contamination.

### Selection of doses of *F. deltoidea* extract

The decoction of *F. deltoidea* is commonly taken twice daily by an adult human, 80–100 mL each time. This volume intake represents about 25–40 g of the extract per day as the yield of the hot water extract of *F. deltoidea* approximately 18% [19]. The dose for the rat was then calculated using the conversion of human equivalent dose (HED) to animal equivalent dose (AED) equation (conversion factor 0.018) based on body surface area [22] as stated below:

$$\mathrm{AED}\ \left(\mathrm{mg}/\mathrm{kg}\right)=\mathrm{HED}\ \left(\mathrm{mg}/\mathrm{kg}\right)\ \mathrm{X}\ \mathrm{conversion}\ \mathrm{factor}$$

Based on this calculation, the possible effective dose of *F. deltoidea* for a rat is ranging between 450 and 720 mg/kg. A toxicity study done by Farsi et al. [21] demonstrated that the LD_50_ of the metabolic extract of *F. deltoidea* was greater than 5000 mg/kg. Considering these factors, three doses were selected to access the effectiveness of *F. deltoidea* to alleviate PCOS symptoms. The three-dose levels are 250 mg/kg (1/20 of LD_50_), 500 (1/10 of LD_50_), 1000 mg/kg (1/5 LD_50_).

### Animals

Female Sprague-Dawley rats (8 weeks old, *N* = 36) weighing between 140 and 170 g were procured from Chenur Sdn. Bhd. Serdang, Selangor. Animals were caged in temperature-controlled steel cages (20–22 °C, 55–65% humidity) with a 12-h light/dark cycle and were feed standard rat chow (Gold Coin Holdings, Kuala Lumpur, Malaysia) and water ad libitum. Rats were allowed to acclimatise for ten days before the experiments. Daily vaginal smears were performed on all rats to evaluate the ovarian function as described by Karateke et al. [23]. Changes in ovulation phases were determined by the number of cornified cells, nucleated epithelial cell leukocytes in vaginal smear morphology. The procedures were performed at 09:00–10:00 AM daily to avoid hormonal fluctuations that may interrupt the cycles. Only those experimental rats displaying two consecutive regular 4-day cycles were included in the study.

### Induction of PCOS rat model

Animals were induced to develop PCOS with LTZ at 1 mg/kg b.wt dissolved in 2 mL/kg/day of saline for 21 days [24]. Approximately 0.5 mL of blood samples were collected before and after PCOS induction from the jugular vein. The samples were centrifuged to obtain serum for testosterone level analysis. Female rats that showed significantly higher levels of testosterone than normal rats with the absence estrus phase in their estrous cycle were selected to represent the PCOS model in this study [25].

### Experiment design

Thirty-six adult female Sprague Dawley rats were randomly assigned into six groups with six rats per each as follows: (i) normal control (NC) received saline at 2 mL/kg/day, (ii) PCOS control (PC) received saline at 2 mL/kg/day, (iii) PCOS rats treated with clomiphene citrate-treated group at 10 mg/kg/day (PCC), (iv) PCOS rats treated with *F. deltoidea* at 250 mg/kg/day group (PFD250), (v) PCOS rats treated with *F. deltoidea* at 500 mg/kg/day group (PFD500), and (vi) PCOS rats treated with *F. deltoidea* at 1000 mg/kg/day group (PFD1000).

Clomiphene citrate (Sigma Chemical Co.) and *F. deltoidea* extract were suspended in 2 mL saline. All treatment was administered via oral gavage for 15 consecutive days [26]. Daily vaginal smears were continuously performed to determine the changes in estrous cycles (absence of estrus stage). All rats were sacrificed at the onset of the diestrus phase [27] as the morphology of female reproductive organs is greatly influenced by the stage of estrous cycles [28].

At the end of the experiment, animals were allowed to fast for 12 h. Fasting blood glucose was determined using Accu-check Advantage II Blood Glucose Monitor (Roche Mannheim, Germany, the upper limit of detection 33.3 mM). Rats were anesthetized with diethyl ether (1.9%) in a large desiccator. The blood was collected from the abdominal aorta into a plain EDTA red-top tube (BD Vacutainer, USA). Serum was kept at − 80 °C for biochemical analysis and hormonal profile. The rats were sacrificed by cutting off the diaphragm. Ovary and uterus were identified, dissected out from the surrounding fats, and measured (weight for ovaries and uteri; length for uteri). The separation of the horns of the uterus from the vagina was done by cutting the uppermost point of the cervix. The right ovary and uterus of each rat were used for antioxidant assay while the left side of these organs was preserved in 10% formalin for histomorphometric analysis.

### Physical parameters measurement

Pre and post body weight and length of the rats were measured at approximately 16:00 clocks during the experiment. Body mass index (BMI), and Lee index were calculated to estimate obesity in animals. The BMI was calculated by dividing the weight (g) by the length (cm^2^) [29]. Body length was defined as the distance from the nose to the anus of rats. Meanwhile, Lee index for each animal was measured by dividing the cube root of the body weight (g) by the naso-anal length (cm^2^) and multiplying the whole expression by 10,000. Rats were considered obese if Lee index value is higher than 310 [30].

### Determination of insulin resistance

Serum insulin concentrations were quantified by Ultra-Sensitive Rat Insulin ELISA kit (Cloud-Clone Corp., Houston, USA) as described by Nurdiana et al. [17]. Homeostatic Model Assessment Insulin resistance (HOMA-IR) was calculated according to Shen et al. [31] as follows:

$$\mathrm{HOMA}-\mathrm{IR}=\frac{\mathrm{Fasting}\ \mathrm{Blood}\ \mathrm{Glucose}\ \left(\mathrm{FBG}\right)\ \mathrm{x}\ \mathrm{Fasting}\ \mathrm{Insulin}\ \left(\mathrm{FINS}\right)}{405}$$

### Analysis of gonadotropins and steroids hormones

The levels of serum testosterone (T), estrogen (E), progesterone (P), luteinizing hormone (LH), and follicle-stimulating hormone (FSH) were measured using Rats ELISA kits procured from Qayee Biotechnology Co., LTD, Shanghai, China. Each hormone was analysed using different kits according to specific antibodies, Horseradish Peroxidase, and the manufacturer’s instructions.

In brief, 10 μL of the serum samples were added (Insulin: 10 μL, Progesterone: 10 μL, Testosterone: 10 μL, Estrogen: 10 μL) into respective pre-coated ELISA plates. The plates were incubated for 1 h. After incubation, the plates were washed five times with 350 μL of diluted washing liquid for 2 min. 50 μL of each chromogen A and chromogen B was further added into each well, gently shaken, and incubated for 10 min. at 37 °C in a dark room as the chromogen is very sensitive to light and may cause sample contamination. Then, 50 μL of Stop solution was added to each well to stop the reaction indicated by a change of colour from blue to yellow. The optical density (OD) was then read at 450 nm wavelength within 15 min. After the stop solution was added. A standard curve was constructed by plotting a graph of the absorbance of each reference standard against its corresponding levels and used to determine each of the hormone levels.

### Measurement of serum lipid

The levels of total cholesterols (TC), triglycerides (TG), low-density lipoprotein cholesterol (LDL), and high-density lipoprotein cholesterol (HDL) were determined using an automatic analyser (Hitachi 911, Boehringer-Mannheim, Germany).

### Measurement of antioxidant and oxidative stress biomarkers

Serum samples were used to assay the activities of antioxidant enzymes, MDA concentrations, and total protein. Bradford method was used to assess the protein concentration in supernatants, with bovine serum albumin as the reference (Sigma Aldrich, St. Louis, MO, USA, Cat. No. B6916).

### Histology analysis

The histological procedures involved in this study were conducted according to the methods mentioned by Mvondo et al. [32]. Ovary and uterus of rats were immersed and fixed in formalin (10%) solution for at least 48 h at room temperature. The selected organs were embedded in paraffin wax, sectioned at 5-μm using Leica RM2245 microtome (Leica Biosystems, Wetzlar, Germany), and air-dried in a vertical position. Ten serial sections of each sample were collected for H&E staining. Histomorphological changes were assessed on microphotographs using a DP70 digital light microscope system (Olympus, Tokyo, Japan) equipped with a camera. The captured image was transferred and analysed with the ImageJ software. Corpus luteum was identified as large, round or irregular glandular structures composed of multiple layers of large granulosa lutein cells. Ovarian cysts were identified as cyst-like structures within the ovary lined by a thin layer of degenerating granulosa cells. Follicles containing an oocyte with a nucleus were counted and described as healthy [23].

### Statistical analysis

All data were expressed as mean ± standard error of the mean (SEM), except data of the estrous cycle. All measurements were assessed for normality using the Kolmogorov-Smirnov test. The unpaired Student t-test was used to compare the initial and final physical parameters. One-way analysis of variance (ANOVA) followed by Duncan multiple comparison post hoc tests was performed to elucidate the statistical differences between the experimental groups. Differences were considered significant at *p* < 0.05.

## Results

### Physical parameters

Table 1 shows the changes in body weight, BMI, and Lee index values during the experimental period. It is noticeable that oral administration of LTZ (1 mg/kg/day) for 21 days to female rats resulted in a significant increase in body weights, BMI, and Lee index values. Higher body weights and Lee index values were maintained in the PC groups throughout the study. Treatment with clomiphene citrate and *F. deltoidea* extract significantly prevented the body weight gain and elevation of Lee Index values in PCOS rats. However, a significant reduction in BMI and Lee Index values was only observed in the PFD500 and PFD1000 groups.

**Table 1:** Changes in body weight (g), BMI (g/cm^2^), and LI value of female PCOS rats

Groups	Initial Body weight (g)	Final Body weight (g)	Initial BMI (g/cm^2^)	Final BMI (g/cm^2^)	Initial Lee’s Index	Final Lee’s Index
NC	200.01 ±5.38^a^	218.66 ±5.27^a^	0.48 ±0.01^a^	0.47 ±0.01^a^	283.15 ±3.97^a^	281.52 ±2.21^a^
PC	268.25 ±9.67^b,x^	298.28 ±7.33^c,y^	0.57 ±0.02^b^	0.59 ±0.02^c^	317.10 ±3.95^b^	311.43 ±6.31^c^
PCC	261.35 ±5.39^b,^	272.15 ±5.81^b^	0.57 ±0.03^b^	0.55 ±0.02^b,c^	299.83 ±1.93^b^	301.35 ±3.54^a,b^
PFD250	260.85 ±3.47^b,^	268.97 ±6.57^b,^	0.56 ±0.01^b^	0.55 ±0.03^b,c^	297.27 ±2.28^b^	297.99 ±5.94^b^
PFD500	260.44 ±2.04^b^	270.78 ±5.53^b^	0.58 ±0.02^b,x^	0.52 ±0.02^a,b,y^	303.85 ±5.17^b,x^	284.58 ±3.89^a,b,y^
PFD1000	267.42 ±6.62^b^	268.42 ±4.67^b^	0.57 ±0.03^b,x^	0.51 ±0.02^a,b,y^	298.03 ±4.49^b,x^	278.73 ±3.40^a,y^

Values are mean ± 1 SD for six rats in each group. Values with different superscripts^a,b,c,d^ in a column differed significantly at p < 0.05 due to treatment effects. Values with different supercripts^x,y^ in a row differed significantly at *p* < 0.05 due to time effects

### Estrous cycle

**Fig. 1:**
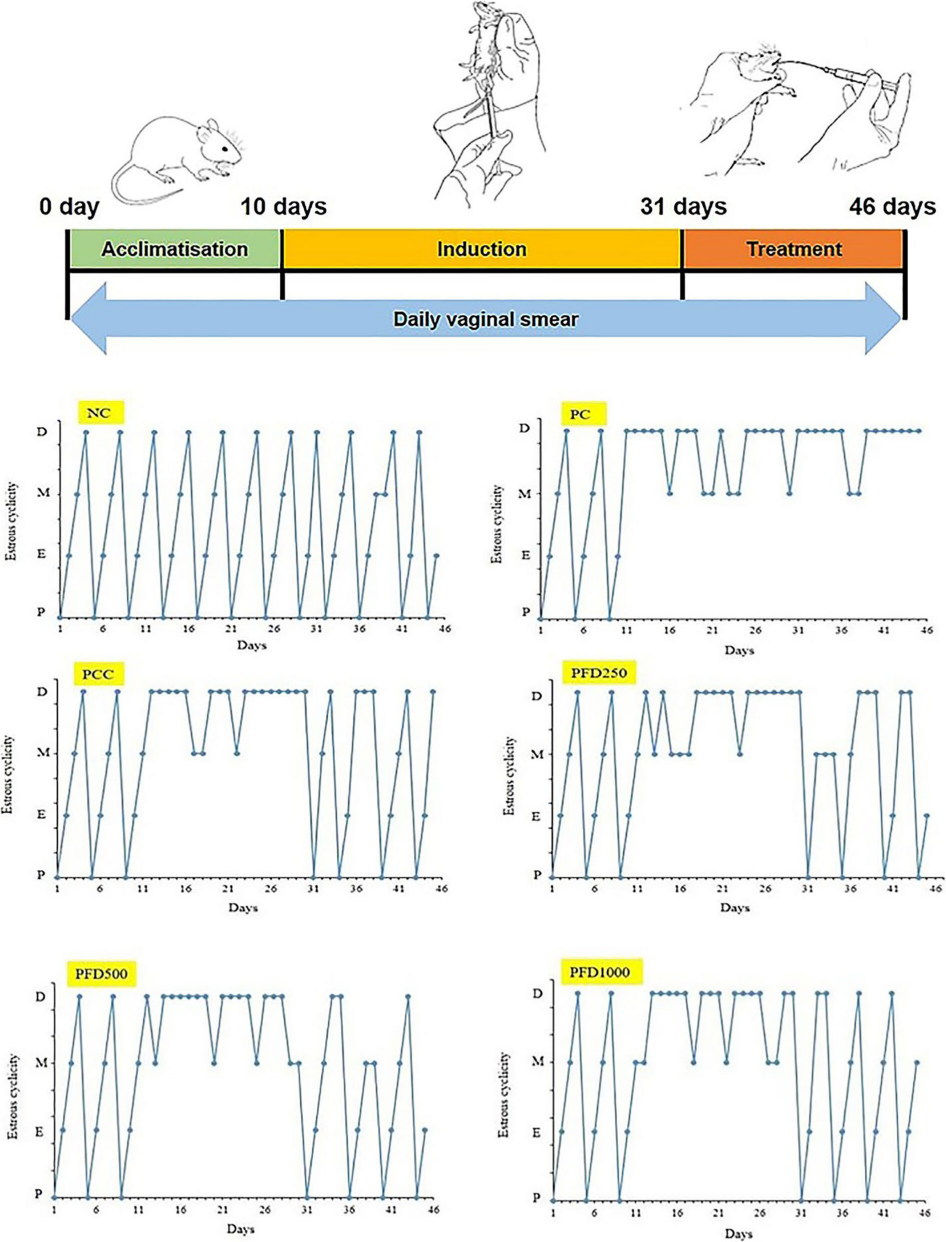
Phases of the estrous cycle in female rats at different days of treatment. NC: normal control; PC: letrozole-induced PCOS control; PCC: clomiphene citrate; PFD250: 250 mg/kg/day of *F. deltoidea*; PFD500: 500 mg/kg/day of *F. deltoidea,* and PFD1000: 1000 mg/kg/day of *F. deltoidea*. Data show the most represented phase of the estrous cycle in each group, *n* = 6. P = proestrous, E = estrous, M = metestrus, and D = diestrous

Figure 1 illustrates the estrous cycle which includes proestrus, estrus, metestrus, and diestrus phases of each experimental group. Animals in the NC group had a regular estrus cycle of 4–5 days throughout the study period. However, the estrous cycle was completely disrupted in all PCOS-like rats and all of them remained mostly at the diestrus or metestrus stages during the induction period (from day 11–31). The PCC, PFD500, and PFD1000 groups displayed improvement in estrous cyclicity from day 32 to 46 (treatment phase). Higher frequency of the estrus phase and less extended diestrus phase were found in comparison to the PC group. Although the PFD250 group showed an improvement in the estrous cyclicity, the presence of an extended diestrus phase was recorded as well as lack of estrus phase throughout the treatment period.

### HOMA-IR analysis

Table 2 showed the fasting blood glucose (FBG), fasting insulin (FINS), and HOMA-IR values of the experimental groups. The PC group had significantly higher values of FBG, FINS, and HOMA-IR than the NC group. Meanwhile, animals in the PCC group showed a significant reduction of the FBG, FINS, and HOMA-IR values in comparison to the PC group. Similar observations were found in the PFD250, PFD500, and PFD1000 groups.

**Table 2:** Changes in the levels of FBG, FINS, and HOMA-IR among the experimental groups

Parameters	Experimental group					
	NC	PC	PCC	PFD250	PFD500	PFD1000
FBG (mg/dL)	103.42 ±1.22^a^	122.82 ±6.21^c^	109.01 ±3.25^a^	118.92 ±2.85^b,c^	115.06 ±1.49^b,c^	114.41 ±2.82^a,b^
FINS (μU/mL)	1.49 ±0.10^a^	2.69 ±0.12^b^	1.68 ±0.18^a^	1.84 ±0.14^a^	1.75 ±0.09^a^	1.65 ±0.12^a^
HOMA-IR	1.46 ±0.04^a^	2.09 ±0.21^c^	1.64 ±0.10^a,b^	1.94 ±0.09^b,c^	1.78 ±0.04^a,b^	1.79 ±0.09^a,b^

Superscripts ^a,b,c^ represent significant difference at *p* < 0.05 among the groups within rows

### Hormonal profiles

As depicted in Table 3, the levels of testosterone, FSH, and LH were increased markedly while the levels of estrogen and progesterone were decreased significantly in the PC group as compared to the NC group. However, all hormonal changes were significantly improved to near normal levels following treatment with clomiphene citrate. *F. deltoidea* improved the hormonal profile of PCOS rats in a dose-dependent manner.

**Table 3:** The effects of *F. deltoidea* on hormonal profiles in LTZ-induced PCOS rats

Hormones	Experimental group					
	NC	PC	PCC	PFD250	PFD500	PFD1000
Testosterone (ng/mL)	93.45 ±2.26^a^	119.11 ±4.86^c^	93.15 ±4.50^a^	110.98 ±4.57^b,c^	98.64 ±4.28^a,b^	94.19 ±3.03^a^
Estrogen (pg/mL)	60.90 ±2.33^b^	40.94 ±7.59^a^	65.37 ±3.43^b^	53.46 ±6.11^a,b^	60.19 ±5.70^b^	64.69 ±4.74^b^
Progesterone (ng/mL)	12.49 ±0.97^c^	8.55 ±0.89^a^	10.13 ±0.50^b,c^	9.23 ±0.99^a,b^	10.06 ±0.47^b,c^	12.15 ±0.81^c^
FSH (mIU/mL)	12.52 ±0.91^a^	18.99 ±0.59^b^	13.88 ±0.80^a^	14.29 ±1.14^a^	13.90 ±1.37^a^	13.14 ±0.74^a^
LH (ng/mL)	24.06 ±1.16^a^	38.82 ±4.56^c^	30.46 ±1.56^a,b^	37.73 ±0.85^c^	33.48 ±1.90^b,c^	29.45 ±1.20^a,b^

Superscripts ^a,b,c^ within a row represent significant difference at *p <* 0.05 among the groups

### Serum lipid profile

The levels of total cholesterol, triglycerides, and LDL-C were increased while the HDL-C decreased significantly in the PC group as compared to the NC groups (Table 4). However, the levels of total cholesterol, triglycerides, and LDL-C were significantly decreased and HDL-C was increased to near-normal levels in all treated animals.

**Table 4:** The effects of *F. deltoidea* on serum lipid profile in LTZ-induced PCOS rats

Lipids Parameters	Groups					
	NC	PC	PCC	PFD250	PFD500	PFD1000
Total cholesterol (mmol/L)	1.872 ±0.11^b,c^	2.603 ±0.10^d^	1.233 ± 0.10^a^	2.052 ± 0.06^c^	1.850 ±0.10^b,c^	1.742 ±0.10^b^
Triglycerides (mmol/L)	0.644 ± 0.05^a^	1.462 ± 0.12^c^	0.603 ± 0.98^a^	1.072 ± 0.11^b^	0.962 ±0.05^b^	0.665 ±0.07^a^
LDL-C (mmol/L)	0.288 ± 0.09^b^	0.435 ± 0.02^d^	0.200 ± 0.17^a^	0.413 ± 0.02^d^	0.378 ±0.03^c,d^	0.335 ±0.02^b,c^
HDL-C (mmol/L)	1.572 ± 0.09^b^	1.227 ± 0.07^a^	1.547 ± 0.06^b^	1.553 ± 0.08^b^	1.588 ±0.08^b^	1.550 ±0.08^b^

Superscripts ^a,b,c,d^ within a column represent significant difference at *p* < 0.05 among the groups

### Antioxidant and oxidative stress activities

The serum SOD and GSH-Px levels reduced while the levels of MDA increased significantly (*p* < 0.05) in the PC group as compared to the NC animals (Table 5). Treatment with clomiphene citrate showed a significant improvement in the SOD, GSH-Px, and MDA levels as compared to the PC group. Similarly, SOD and GSH-Px levels were also increased and MDA levels decreased in the PFD500 and PFD1000 groups.

**Table 5:** The effect of *F. deltoidea* on antioxidant enzymes and lipid peroxidation in LTZ-induced PCOS rats

Groups	Antioxidant enzymes and lipid peroxidation levels		
	SOD (U/mL)	GSH-Px (U/L)	TBARS (μmol, MDA)
NC	1.90 ± 0.15^b^	65.47 ± 1.622^d^	1.69 ± 0.14^a^
PC	0.78 ± 0.09^a^	40.44 ± 3.52^a^	4.03 ± 0.34^c^
PCC	1.66 ± 0.27^b^	52.61 ± 3.68^b,c^	2.22 ± 0.37^a^
PFD250	0.85 ± 0.17^a^	43.02 ± 3.31^a,b^	3.43 ± 0.34^b,c^
PFD500	1.37 ± 0.24^a,b^	48.58 ± 2.74^a,b,c^	2.72 ± 0.39^a,b^
PFD1000	1.70 ± 0.29^b^	55.27 ± 5.46^c,d^	2.09 ± 0.29^a^

Superscripts a,b,c within a column represent significant difference at p < 0.05 among the groups

### Ovarian Histomorphometric changes

**Fig. 2:**
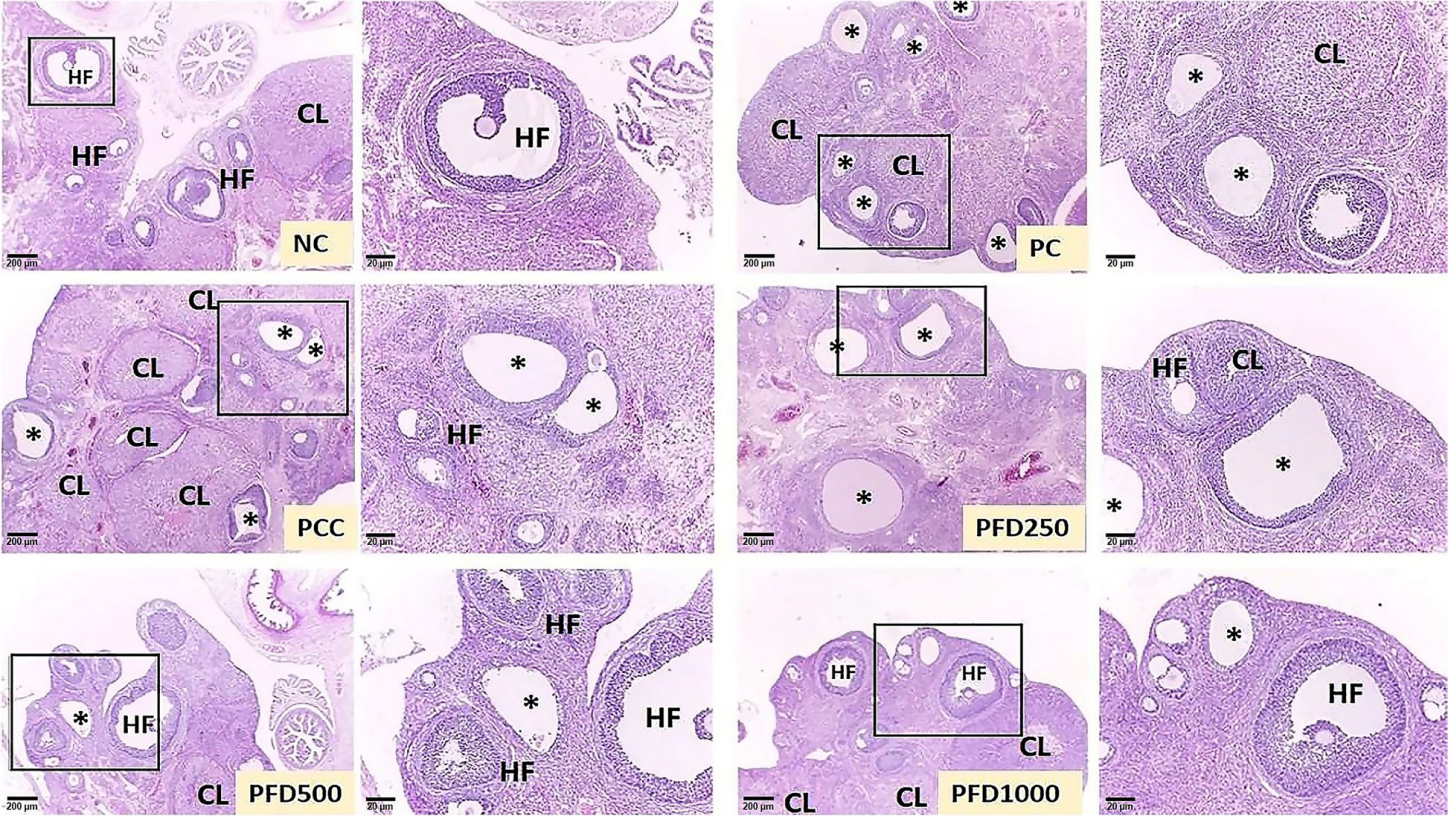
Photomicrographs of the representative ovarian cross-section at different magnification from all experimental groups. NC group displaying normal histological appearance with corpus luteum (CL) and several healthy follicles (HF). Ovarian cross-sections from the PC group showing many cystic follicles (*) with lesser CL and HF. PCC group showing reduced cystic follicles (*) with abundant CL and HF. PFD250 group was associated with fewer cystic follicles (*) and the presence of HF and CL. PFD500 and PFD1000 groups showing vast improvement in the histological structure by the absence of cystic follicles (*) and many HF and CL which were almost comparable with the NC group. The cross-sections were stained with heamatoxylin-eosin (H&E) with 4X and 40X magnification. NC: normal control; PC: letrozole-induced PCOS control; PCC: clomiphene citrate; PFD250: 250 mg/kg/day of *F. deltoidea*; PFD500: 500 mg/kg/day of *F. deltoidea,* and PFD1000: 1000 mg/kg/day of *F. deltoidea*

Transverse sections of ovaries from the NC group showed normal histological morphology with several healthy follicles at different stages of oocyte development and the presence of corpus luteum (Fig. 2). However, fewer numbers of corpus luteum and multiple follicular ovarian cysts were observed in the PC group. The number of corpus luteum was significantly decreased by 56.9% while total numbers of the cystic follicles significantly increased by 608.3% in the PC group as compared to the NC group (Table 6). LTZ treatment also resulted in a significant reduction in ovary weight. In contrast, the cystic follicle count was significantly reduced while the numbers of corpus luteum and healthy follicles were significantly increased in all treatment groups.

**Table 6:** Changes of ovarian morphological parameters in all experimental groups

Groups	Ovary weight (g)	Total numbers of cystic follicles	Numbers of corpus luteum	Numbers of healthy follicles
NC	0.20 ± 0.02^b^	1.20 ± 0.20^a^	5.40 ± 0.51^c,d^	10.40 ± 0.50^c^
PC	0.11 ± 0.01^a^	8.50 ± 0.43^e^	2.33 ± 0.33^a^	4.17 ± 0.48^a^
PCC	0.17 ± 0.02^b^	2.17 ± 0.31^a,b^	6.07 ± 0.37^d^	9.50 ± 0.62^c^
PFD250	0.20 ± 0.02^b^	7.00 ± 0.36^d^	3.17 ± 0.40^a,b^	4.33 ± 0.42^a^
PFD500	0.20 ± 0.01^b^	2.83 ± 0.47^b^	4.16 ± 0.60^b,c^	6.33 ± 0.42^b^
PFD1000	0.22 ± 0.03^b^	4.20 ± 0.37^c^	4.20 ± 0.37^b,c^	7.20 ± 0.58^b^

Superscripts ^a,b,c^ within a column represent significant difference at *p* < 0.05 among the groups

### Uterine Histomorphometric changes

**Fig. 3:**
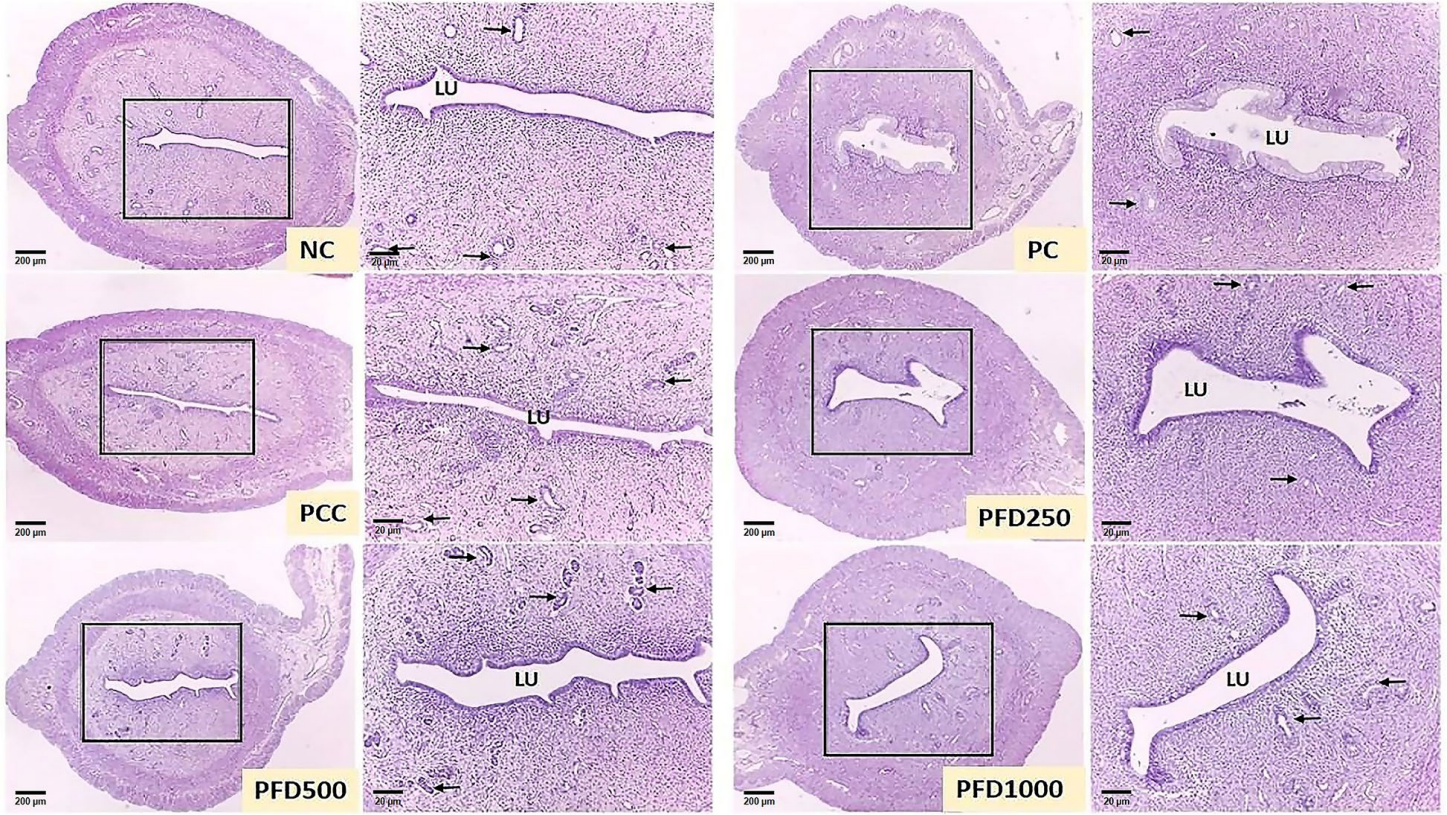
Photomicrographs of the representative uterine cross-section at different magnification from all experimental groups. NC group showing normal histological appearance with normal endometrium wall (red arrow), and endometrial gland (EG) (black arrow). PC group displaying a thin endometrium wall with lesser EG. PCC group showing visible endometrium wall with abundant EG. PFD250 group showing less visible endometrium wall with several. PFD500 and PFD1000 displaying an improvement in the histological structures by the presence of EG, visible endometrium wall, and thick uterus which were almost comparable with the NC group. The cross-sections were stained with heamatoxylin-eosin (H&E) with 4X and 40X magnification. NC: normal control; PC: letrozole-induced PCOS control; PCC: clomiphene citrate; PFD250: 250 mg/kg/day of *F. deltoidea*; PFD500: 500 mg/kg/day of *F. deltoidea,* and PFD1000: 1000 mg/kg/day of *F. deltoidea*

Figure 3 illustrates the photomicrographs of uterine tissues from each experimental group. Smaller uterus size and endometrial thicknesses were observed in the PC group. Morphometric measurements in the PC group revealed that the length of the uterine, endometrium thickness, and numbers of the endometrial gland, were significantly decreased by 25.9, 36.1, and 47.7%, respectively compared to the NC group (Table 7). However, the PCC, PFD500, and PFD1000 groups displaying an improvement in the histological structures. The endometrium wall and endometrial gland were visible following these treatments. The mean endometrium thickness was normalized in the PFD1000 while treatment with clomiphene citrate normalized the numbers of the endometrial gland in PCOS rats. A significant increase (*p* < 0.05) in uterus weight was also observed in the PCC and PFD1000 groups.

**Table 7:** Changes of uterine morphological parameters in all experimental groups

Group	Uterine weight (g)	Uterus Length (μm)	Endometrium Thickness (μm)	Numbers of Endometrial gland (n)
NC	0.46 ± 0.03^b,c^	183.05 ± 3.07^c,d^	135.79 ± 2.91^c^	11.80 ± 0.66^b^
PC	0.28 ± 0.02^a^	135.67 ± 2.79^a^	86.71 ± 1.43^a^	6.17 ± 0.31^a^
PCC	0.44 ± 0.06^b,c^	200.00 ± 2.79^d,e^	133.07 ± 2.58^b,c^	12.00 ± 0.58^b^
PFD250	0.35 ± 0.02^a,b^	158.72 ± 3.33^b^	115.37 ± 8.13^b^	6.67 ± 0.66^a^
PFD500	0.37 ± 0.05^a,b,c^	172.25 ± 2.98^b,c^	123.85 ± 4.91^b,c^	12.50 ± 0.56^b^
PFD1000	0.49 ± 0.06^c^	210.95 ± 19.75^e^	139.80 ± 11.51^c^	14.40 ± 0.68^c^

Superscripts ^a,b,c,d,e^ within rows represent significant difference at *p* < 0.05 among the groups

## Discussion

The present study demonstrated that LTZ-induced PCOS rats exhibit the clinical and biochemical characterizations of women with PCOS. In line with previous observations, our results showed PCOS-like conditions in rats including an abnormal estrus cyclicity, elevated body weight, insulin resistance, total cholesterol, triglycerides, LDL-C, MDA, LH, FSH, and testosterone levels [33]. The animals also had lower HDL-C, estrogen, progesterone, SOD, and GSH-Px levels than the normal control group. We demonstrated, for the first time, that treatment with *F. deltoidea* at 500 and 1000 mg/kg/day can ameliorate PCOS symptoms in rats by improving insulin resistance, antioxidant activity, and hormonal balance. We also showed that *F. deltoidea* significantly decreases the number of cystic follicles, increases the number of corpus luteum, and normalizes the endometrium thickness.

Treatment with LTZ to adult rats for 21 days increased body weight, BMI, Lee’s index (Table 1), and resulted in disruption of the estrous cycle (Fig. 1). Lee’s index values of the PC group were higher than 310 throughout the experimental period, indicating the efficacy of LTZ in the induction of PCOS. A significant increase between the initial and final measurement of BMI further confirms the overweight and obesity incidence in PCOS rats. Similar findings have been reported in different animal models of PCOS [34–36]. It is important to note that *F. deltoidea* treatment at 500 and 1000 mg/kg/day for up to 15 days significantly reduced BMI and Lee’s Index values in PCOS rats. A small weight loss of approximately 5% can improve insulin resistance, hormone levels, menstrual cycles, and infertility associated with PCOS [37, 38]. Indeed, lower HOMA-IR values were found in the PFD500 and PFD1000 groups (Table 2), suggesting the insulin-sensitizing activity of *F. deltoidea*. Animals in these groups were also associated with improvement in estrous cyclicity during the treatment phase. These findings were consistent with the notion that insulin sensitiser may improve menstrual cyclicity and ovulation in PCOS [39].

We demonstrated that *F. deltoidea* treatment at 500 and 1000 mg/kg/day significantly reduced the concentrations of testosterone, FSH, and LH as well as increased estrogen and progesterone to near-normal levels in PCOS rats (Table 4). These results imply that *F. deltoidea* exhibited anti-androgenic and estrogenic properties in PCOS rats that in turn can explain the suppression of FSH and LH. A similar finding has been reported by Nur Ajeerah et al. [16]. Supporting this view, the presence of catechin, gallocatechin, and epigallocatechin have been reported by Haida et al. [40]. Catechin is known to suppress appetite, reduce food consumption, and is responsible for the reduction in testosterone level [41, 42]. Meanwhile, epigallocatechin and apigenin were proven to inhibit 17β-HSD, 3β-HSD enzymes, and P450 activity that lead to inhibitory effects on testosterone production. However, Rocha et al. [43] demonstrated that normalization of testosterone levels did not improve BMI, glucose, or lipid metabolism in postmenopausal hyperandrogenism rats. It should be mentioned that the effects of serum androgen normalization are different in reproductive and non-reproductive status. It has also been shown that therapy aimed at reducing androgen over-production failed to ameliorate insulin resistance in PCOS [44]. Further analyses are, therefore, needed to assess the biological outcomes following *F. deltoidea*.

Lipid disturbances are the most common metabolic abnormality in PCOS. Our current results showed that the administration of LTZ for 21 days was not only able to develop PCOS symptoms similar to those occurring in humans but also affect the serum lipid profiles (Table 4). A similar finding has been reported by Ndeingang et al. [45]. Nevertheless, Wasan et al. [46] provide evidence that LTZ did not significantly alter serum lipid profile. This discrepancy can be justified by the fact that LTZ only has a good short-term tolerability profile [47]. Continuous exposure to LTZ has been demonstrated to cause significant changes in lipid profile [48]. It was found that *F. deltoidea* restored the levels of total cholesterol, triglycerides, LDL-C, and HDL-C to normal levels in PCOS rats that had improvement in hormonal profile and the estrous cycle. The data support that the relationship between lipid profile and sex hormones [49, 50]. These results are also compatible with earlier studies demonstrating that *F. deltoidea* can reverse the abnormalities in the lipid profile of diabetic rats [51] and adults with pre-diabetes [52],

Lipid peroxidation and antioxidants were further evaluated to understand the potential of *F. deltoidea* in treating PCOS. We found that the PCOS rats had higher serum MDA and lower endogenous antioxidant enzymes (SOD and GSH-Px) levels than normal control (Table 5), indicating oxidant-antioxidant imbalance occurred. A disturbance in the antioxidant-prooxidant balance has been reported to induce pathological consequences in oocyte maturation, ovulation, fertilization, implantation, and embryo development [53]. In contrast, PCOS animals in the PFD500 and PFD1000 groups had a significantly lower level of MDA but higher SOD and GSH-Px activities. These findings in agreement with data which were reported the prospect of oxidative stress modulator-natural antioxidants as therapeutic interventions for managing PCOS [54]. However, an increase in SOD activity has also been reported in women with PCOS [55]. A higher level of SOD activity and reduced levels of glutathione peroxidase has been shown to disrupt the efficiency of ROS scavenging in the follicular environment [56]. Therefore, histomorphometric analysis of the ovary and uterine tissues are required to confirm these results.

PCOS has been demonstrated to induce histo-architectural changes in the ovary [57] and uterus [58] of rats. In agreement with previous studies on PCOS animal models, fewer numbers of corpus luteum and multiple follicular ovarian cysts were observed in the PC group [59, 60]. Reduction in ovarian weight, uterine length and weight, endometrium thickness, and numbers of the endometrial gland are consistent with decreasing levels of testosterone, FSH, LH, and antioxidants activities. Our study provides additional support for the association between reproductive hormone levels and gonadal morphology in rats with PCOS. Importantly, significant increases in the numbers of corpora lutea, ovarian and uterine weight, endometrium thickness, and numbers of the endometrial gland, together with a decrease in the numbers of cystic follicles were found in the PCC, PFD500, and PFD1000 groups. The appearance of corpora lutea suggest that these animals have ovulated. Higher levels of progesterone obtained in these groups confirmed that ovulation has occurred. It is important to note that the management of PCOS is aimed mainly at restoring ovulation.

Taken together, our results showed that *F. deltoidea* extract effectively ameliorates biochemical, hormonal, and histomorphometric changes to levels comparable with those reported in the clomiphene citrate-treated rats. It may be suggested that *F. deltoidea* extract promotes adiponectin, a protein hormone that modulates several metabolic processes, including glucose regulation and fatty acid oxidation [61]. These results support the hypothesis that a combination of lipid-lowering with insulin-sensitizing agents would achieve better therapeutic effects in the treatment of PCOS [62]. Therefore, future studies are required to determine the value of serum adiponectin levels in the PCOS rat model.

## Conclusion

We conclude that *F. deltoidea* can reverse the symptoms of PCOS in female rats by improving insulin sensitivity, lipid profile, antioxidant activity, hormonal balance. These findings improve our current understanding of the potential use of *F. deltoidea* for the treatment of PCOS.

## Data Availability

All data generated or analyzed during this study are included in this manuscript.

## Abbreviations

ANOVA: Analysis of variance
ELISA: Enzyme-linked immunosorbent assay
LTZ: Letrozole
GSH-PX: Glutathione peroxidase enzyme
H&E: Hematoxylin and eosin
PCOS: Polycystic ovarian syndrome
SOD: Superoxide dismutase enzyme
LDL: Low-density lipoprotein cholesterol
MDA: Malondialdehyde
LH: Luteinizing hormone
FSH: Follicle-stimulating hormone
NC: Normal control
PC: Letrozole-induced PCOS control
PCC: Clomiphene citrate
PFD250: 250 mg/kg/day of *F. deltoidea*
PFD500: 500 mg/kg/day of *F. deltoidea*
PFD1000: 1000 mg/kg/day of *F. deltoidea*
